# Large-Scale Network Coupling with the Fusiform Cortex Facilitates Future Social Motivation

**DOI:** 10.1523/ENEURO.0084-17.2017

**Published:** 2017-10-11

**Authors:** Amanda V. Utevsky, David V. Smith, Jacob S. Young, Scott A. Huettel

**Affiliations:** 1 Center for Cognitive Neuroscience, Duke University, Durham, NC 27708; 2 Department of Psychology and Neuroscience, Duke University, Durham, NC 27708; 3 Department of Psychology, Temple University, Philadelphia, PA 19122; 4 Pritzker School of Medicine, University of Chicago, Chicago, IL 60637

**Keywords:** default-mode network, effective connectivity, executive control, faces, goal-directed behavior

## Abstract

Large-scale functional networks, as identified through the coordinated activity of spatially distributed brain regions, have become central objects of study in neuroscience because of their contributions to many processing domains. Yet, it remains unclear how these domain-general networks interact with focal brain regions to coordinate thought and action. Here, we investigated how the default-mode network (DMN) and executive control network (ECN), two networks associated with goal-directed behavior, shape task performance through their coupling with other cortical regions several seconds in advance of behavior. We measured these networks’ connectivity during an adaptation of the monetary incentive delay (MID) response-time task in which human participants viewed social and nonsocial images (i.e., pictures of faces and landscapes, respectively) while brain activity was measured using fMRI. We found that participants displayed slower reaction times (RTs) subsequent to social trials relative to nonsocial trials. To examine the neural mechanisms driving this subsequent-RT effect, we integrated independent components analysis (ICA) and a network-based psychophysiological interaction (nPPI) analysis; this allowed us to investigate task-related changes in network coupling that preceded the observed trial-to-trial variation in RT. Strikingly, when subjects viewed social rewards, an area of the fusiform gyrus (FG) consistent with the functionally-defined fusiform face area (FFA) exhibited increased coupling with the ECN (relative to the DMN), and the relative magnitude of coupling tracked the slowing of RT on the following trial. These results demonstrate how large-scale, domain-general networks can interact with focal, domain-specific cortical regions to orchestrate subsequent behavior.

## Significance Statement

A diverse set of behaviors, from normal to pathologic, has been linked to the responses of large-scale functional networks. Yet, it remains unclear how these domain-general networks shape subsequent processing in cortical regions with domain-specific function. Here, we examine how two such networks, the default-mode network (DMN) and executive control network (ECN), connect functionally with other cortical regions to alter performance in an incentive-compatible task. We found that differential coupling between a prototypical face processing region and DMN and ECN tracked subsequent improvements in performance to social stimuli. Our approach allowed us to examine direct coupling with functional networks to future behavior, providing a significant step forward in understanding how large-scale networks coordinate thought and action.

## Introduction

Since the discovery of functionally correlated brain regions (Biswal et al., 1995), large-scale functional networks have been considered fundamental features of brain activity (Gusnard and Raichle, 2001; Beckmann et al., 2005; Behrens and Sporns, 2012). These networks are highly reliable across large samples of participants (Biswal et al., 2010; Smith et al., 2013) and are thought to reflect intrinsic properties of brain organization. Many of these networks reflect sensory and perceptual processes instantiated within visual or auditory regions (Smith et al., 2009). Still others contribute broadly to cognitive processing across stimulus and task domains, including the default-mode (DMN), executive control (ECN), and frontoparietal networks (Dosenbach et al., 2007; Smith et al., 2009). These functional networks have been linked to various aspects of behavior including demographic variables (Filippini et al., 2009; Cowdrey et al., 2014), traits (Shannon et al., 2011; Kucyi et al., 2014), and cognitive states (Smith et al., 2009; De Havas et al., 2012).

Yet, for effective behavior in a particular task, these large-scale domain-general networks must alter ongoing task-specific processing in focal brain regions. To claim that such interactions (i.e., between large-scale networks and specific brain regions) are critical for behavior change, several conditions should be met. First, a large-scale network should be identifiable during task performance independently of other concurrent activation; that is, networks should be able to be extracted regardless of other processes occurring in focal brain regions that might contribute to that task (i.e., without relying on seed-based analyses; Cole et al., 2010; Smith et al., 2014b). Second, the coupling between a given network and a given focal brain region should systematically vary across task conditions according to the relative engagement of the task (Friston et al., 1997; O’Reilly et al., 2012). Third, those changes in coupling (e.g., effective connectivity; Friston, 2011) should predict the characteristics of subsequent behavior, to provide evidence that the coupling contributes to effective task performance. If these conditions are met, there would be strong evidence that coupling between a functional network and a focal brain region contributes to a specific cognitive process.

In the current study, we examined effective connectivity between large-scale networks and focal brain regions while subjects played a reaction times (RTs) game to receive social and nonsocial rewards in a modified monetary incentive delay (MID) task (Knutson et al., 2000). We have previously used this task to produce meaningful variability in reaction time behavior that reflects relative motivation (Clithero et al., 2011). We focused on the DMN and ECN since both have been linked to task performance, engagement, and other markers of executive function and preparatory behavior. Behaviorally, we found that participants exhibited slower response times (RTs) subsequent to social relative to nonsocial trials, reflecting a change in motivation according to social stimulus type. We investigated whether coupling with DMN and ECN contributed to this subsequent-RT effect by combining independent components (ICA) and psychophysiological interactions (PPIs) analyses. This network-based PPI (nPPI) pipeline allowed us to examine how DMN and ECN contribute to other cortical regions to shape subsequent motivated behavior up to several seconds later. Strikingly, we found that DMN and ECN differentially coupled with a region in the fusiform gyrus (FG) when subjects viewed the social rewards, and that changes in this coupling tracked the effect of stimulus type on subsequent RT. This region of the FG is consistent with the functionally-defined fusiform face area (FFA), a region classically associated with face processing (Kanwisher et al., 1997; McCarthy et al., 1997).

These findings highlight functional network interactions that guide subsequent changes in reaction time behavior. While the cognitive mechanisms underlying the behavioral effect of previous stimulus type on subsequent reaction time require future research, we propose that this effect may be driven by increased attentional interference of social rewards relative to nonsocial rewards or a subsequent change in task motivation. Collectively, these results indicate that functional networks associated with goal-directed behavior can interact with focal brain regions to support future motivated behavior.

## Materials and Methods

### Participants

A group of 50 self-reported heterosexual males completed the task (mean age: 23.8 years, range 18–32); this sample size was established before data collection. All participants were screened before data collection to rule out prior or current psychiatric or neurologic illness. We excluded four participants because of data quality issues (see below, Preprocessing), and we excluded five participants because of a malfunctioning button box in the scanner. These exclusions left a final sample of 41 participants (mean age: 24.1 years, range: 18–32). All participants gave written informed consent as part of a protocol approved by the Institutional Review Board of Duke University Medical Center.

### Stimuli and tasks

The experiment consisted of four components: (1) a training session outside the scanner; (2) a modified MID task (Knutson et al., 2000, 2001); (3) a passive viewing task with results previously published (Young et al., 2015); and (4) two additional postscan rating tasks. All participants received variable cash payment depending on their performance.

**Figure 1.**
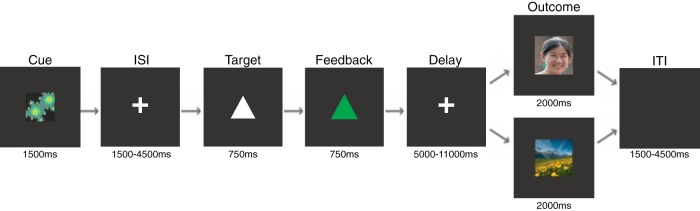
Task design. Fifty heterosexual men performed a modified MID task which examined timed responses to view social and nonsocial images as rewards (faces and landscapes, respectively). Each trial of the task began with an abstract image presented as a cue for 1500 ms indicating the potential reward for that trial. Following the cue, a fixation cross appeared for a variable duration (interstimulus interval, 1500–4500 ms), followed by a target (a white triangle) that appeared on the screen for 750 ms; participants responded by pressing a button box with their right index finger before the target disappeared. After the target, a feedback image (a colored triangle) appeared for 750 ms; the feedback image was colored green if the participants’ response was sufficiently fast on the trial (a win), and was blue if the response was too slow (a loss). After the feedback image disappeared, the participants waited a delay period that varied according to the cue and whether the subject won or lost that trial. Lastly, the reward image (either a face or a landscape) was shown for 2000 ms. Consent was provided for the use of the image in this figure.

In advance of the neuroimaging session, participants memorized the associations of four fractal images with different potential rewards (example in Fig. 1). On the day of the scan, subjects were tested on the fractal images’ associations to ensure they remembered the meaning of each image; if participants identified every association correctly on the first attempt (*n* = 31), they received an extra $5 in addition to their study compensation. All participants successfully learned the image associations before entering the scanner.

Each trial of the modified MID task began with a fractal image presented as a cue for 1500 ms (Fig. 1). The different cues indicated the different potential benefits of a fast response on that trial: (1) reducing the delay until the presentation of a face image of a variable attractiveness rating (social delay; SD); (2) increasing the attractiveness of a face image presented after a variable duration (social attractiveness; SA); (3) reducing the delay until the presentation of a landscape image of a variable attractiveness rating (nonsocial delay; ND); and (4) increasing the attractiveness of a landscape image presented after a variable duration (nonsocial attractiveness; NA).

Following the cue, a fixation cross appeared for a variable duration (interstimulus interval; 1500–4500 ms), followed by a target (a white triangle) that appeared on the screen for 750 ms. Participants then responded by pressing a button box with their right index finger before the target disappeared. A feedback shape then appeared for 750 ms; that shape was colored green if the participant’s response was sufficiently fast (a win), and was colored blue if the response was too slow (a loss). After the feedback image disappeared, participants waited a delay period that varied according to the cue and whether the subject won or lost that trial. Lastly, the reward image (either a face or a landscape) was shown for 2000 ms, and there was an intertrial interval (ITI) that was jittered between 1500 and 4500 ms (with the 2000-ms reward image duration, the minimum and maximum time delays between reward onset and subsequent cue onset was 3500 and 6500 ms, respectively).

Each run of the modified MID task consisted of 36 trials, nine images of each SD, SA, ND, and NA in randomized order. Across all runs included in the analyses, the four types of transitions (e.g., social followed by social, social followed by nonsocial, etc.) each occurred on ∼25 ± 1% of trials, reflecting effective randomization of stimulus types across trials. Social images were photographs of young adult women and were cropped to show only the face (images were drawn from Smith et al., 2010). Nonsocial images were photographs of landscapes, and did not contain any body or facial features. All images were resized to uniform dimensions. Attractiveness (high/medium/low) of images was determined by ratings by an independent group. Additionally, performance thresholds were determined independently for each subject by an algorithm that adapted the task difficulty so that accuracy would be ∼60% (observed hit rate = 60.7%).

After completing the four runs of the modified MID task, participants completed an unrelated passive-viewing task described elsewhere (Young et al., 2015). Following this second scanner task, participants completed two tasks outside the scanner. In the first task, participants rated the attractiveness of each image they viewed in the modified MID task on a scale of 1–7. Each participant’s highest-rated face and landscape images were then used in a second task involving forced choices between a landscape image and a face image presented simultaneously.

All tasks were programmed and displayed using the Psychophysics Toolbox (version 3; Brainard, 1997) for MATLAB (Mathworks).

### Behavioral analysis

Behavioral data were analyzed using MATLAB and IBM SPSS Statistics 20. A RT difference was calculated for each participant by subtracting their mean RT on the trials subsequent to nonsocial trials from their mean RT on trials subsequent to social trials. Consequently, positive RT differences indicate shorter RTs subsequent to nonsocial trials (relative to social trials), whereas negative differences indicate shorter RTs subsequent to social trials (relative to nonsocial trials).

To control for factors other than prior stimulus category, such as attractiveness of the prior trial’s reward image, the prior trial’s RT, and the subsequent trial’s stimulus type, we incorporated these factors into a general linear model (GLM) for each subject. The GLM included the following as regressors: (1) stimulus type on previous (*t*-1) trial; (2) RT on previous trial; (3) attractiveness rating of the reward image of the previous trial; and (4) stimulus type on current trial *t*. This analysis indicates the specific influence of each regressor on the current trial’s RT, and allowed us to examine the unique influence of the previous trial’s stimulus category (social or nonsocial) on current RT. In our analyses, positive stimulus type β weights reflect slower RTs on or following social trials, whereas negative stimulus type β weights reflect faster RTs on or following social trials.

### Image acquisition

Neuroimaging data were collected using a General Electric MR750 3.0 Tesla scanner equipped with an 8-channel parallel imaging system. We used a T_2_*-weighted spiral-in sensitivity encoding sequence (SENSE factor = 2), with slices parallel to the axial plane connecting the anterior and posterior commissures [repetition time (TR): 1580 ms; echo time (TE): 30 ms; matrix: 64 × 64; field of view (FOV): 243 mm; voxel size: 3.8 × 3.8 × 3.8 mm; 37 axial slices; flip angle: 70 degrees]. The first eight volumes of each run were removed to allow for magnetic stabilization. We additionally acquired whole-brain high-resolution anatomic scans (T1-weighted FSPGR sequence; TR: 7.58 ms; TE: 2.93 ms; matrix: 256 × 256; FOV: 256 mm; voxel size: 1 × 1 × 1 mm; 206 axial slices; flip angle: 12 degrees) to allow for coregistration and normalization.

### Preprocessing

Our preprocessing used tools from the FMRIB Software Library package (FSL version 4.1.8; http://www.fmrib.ox.ac.uk/fsl/; Smith et al., 2004; Woolrich et al., 2009). We corrected for head motion by realigning the time series to the middle volume (Jenkinson and Smith, 2001), and then removed nonbrain material using a brain extraction tool (Smith, 2002). We then corrected intravolume slice-timing differences using Fourier-space phase shifting to align to the middle slice (Sladky et al., 2011). After spatially smoothing the image using a 5-mm full-width-half-maximum isotropic Gaussian kernel, we applied a high-pass temporal filter with a 100-s cutoff, and we normalized each 4-dimensional dataset to the grand-mean intensity using a single multiplicative factor. Lastly, we spatially normalized the functional data to the Montreal Neurologic Institute (MNI) Template avg152 T1-weighted template (3-mm isotropic resolution) using a 12-parameter affine transformation implemented in FLIRT (Jenkinson and Smith, 2001).

As part of our preprocessing and quality control, we additionally examined three partially correlated measures of quality assurance: signal-to-fluctuation-noise ratio (SFNR; Friedman and Glover, 2006), volume-to-volume head motion, and number of motion spikes within the time series (motion spikes were identified by evaluating the root-mean-square-error of each volume relative to the middle time point). Measures on each metric were considered outliers if they exceeded the 75th percentile plus the value of 150% of the interquartile range (i.e., a standard boxplot threshold); runs that were identified as outliers were excluded from further analyses. Additionally, any participant who had fewer than two good runs (out of four total runs) was excluded from further analyses. These criteria eliminated four participants.

### Neuroimaging analysis

To best meet the conditions for identifying task-specific interactions between large-scale networks and focal brain regions (see Introduction), our neuroimaging analyses proceeded in two phases, each described in a separate section below. First, we used ICA (Beckmann and Smith, 2004) and spatial regression (Filippini et al., 2009) to identify the large-scale neural networks of interests (DMN and ECN) and to examine the networks’ levels of activation over the course of each run. Second, we used generalized nPPI models (adapted from McLaren et al., 2012) to identify brain regions whose coupling with the ECN and DMN changed as a function of the effect of stimulus type on subsequent RT. Importantly, this ICA-based nPPI approach follows the logic of region of interest (ROI)-based PPI analyses, with the critical difference of examining connectivity with data-driven large-scale neural networks instead of a specific seed region. Critically, we note that this nPPI pipeline allowed us to test the three necessary conditions for inferences that functional networks alter task-specific processing in focal brain regions.

### Identifying large-scale functional networks

We used FSL’s Multivariate Exploratory Linear Decomposition into Independent Components (MELODIC) version 3.10 to identify large-scale functional networks in the neuroimaging data (Beckmann and Smith, 2004). MELODIC ICA was implemented using temporal concatenation, which looks for common spatial patterns of components across participants’ data without assuming a specific or common time course across all participants; we note that previous research has also used temporal concatenation-based ICA on task-based fMRI data (Utevsky et al., 2014; Young et al., 2015). The preprocessed data were whitened and projected into a 25-dimensional subspace (Ray et al., 2013; Utevsky et al., 2014). The whitened data were decomposed into sets of vectors describing the temporal and spatial signal variation, using a fixed-point iteration technique to optimize non-Gaussian spatial source distribution (Hyvärinen, 1999). The estimated component maps were then thresholded by dividing the maps by the standard deviation of the residual noise, then fitting a Gaussian-γ mixture model to the histogram of the normalized intensity values (Beckmann and Smith, 2004). This first step provided a data-driven means to identify functional networks present during task performance; this allowed us to meet the first condition for identifying task-specific interactions between large-scale networks and specific brain regions.

All unthresholded spatial maps from the ICA were then submitted to a spatial regression (part of FSL’s dual regression analysis) to estimate the time courses of each network (Filippini et al., 2009; Leech et al., 2011). In this analysis, spatial maps are regressed onto each participant’s functional data, resulting in a matrix of T (time points) × C (components) β coefficients that characterize each subject’s time courses for each network.

### Characterizing reward-related network connectivity and activation

We assessed task-dependent network coupling using a generalized nPPI model. The generalized PPI, in contrast to a standard PPI, computes a separate PPI term for each task condition. This approach has been shown to yield more accurate estimates of how connectivity varies as a function of psychological context (McLaren et al., 2012). The generalized nPPI analysis was conducted using FMRI Expert Analysis Tool (FEAT) version 5.0.1.

The run-level model included six task regressors: social cue (duration = 1.5–4.5 s), nonsocial cue (duration = 1.5–4.5 s), hits (duration = 0.75 s), misses (duration = 0.75 s), social reward outcome (duration = 2 s), and nonsocial reward outcome (duration = 2 s). We additionally included time courses of both the DMN and ECN that were produced by the spatial regression. Because of the minimum ITI of 1500 ms and the potential confound of the cue presentation occurring between the outcome and subsequent target phases of the task, we examined whether there was any collinearity between the outcomes and cues across the runs included in our analysis. For each run included in our analyses, we calculated the correlation values between the face and land outcome regressors and the face and land cue regressors, and then averaged these correlation values across all runs. The mean correlation values between face or land cues and face or land outcomes ranged from *r* = −0.25 to *r* = −0.17 (minimum *r* = −0.28; maximum *r* = −0.10), indicating that our run-level analyses were able to isolate and model cues and outcomes independently, with minimal collinearity between regressors.

For our network interaction analysis, nPPI regressors were formed by multiplying the DMN and ECN time courses (zeroed to the mean of the time course), separately, by the social outcome and nonsocial outcome regressors (zeroed to the minimum value of the task time course; McLaren et al., 2012); this yielded four nPPI regressors: (1) DMN*social; (2) DMN*nonsocial; (3) ECN*social; and (4) ECN*nonsocial. To control for motion in the scanner, we additionally included motion spikes and motion parameters as regressors. Lastly, to control for the influence of other networks and potential artifacts on our generalized nPPI, we included the time courses of the remaining 23 components from the ICA. The nPPI analysis allowed us to examine task-specific coupling between ECN and DMN with other regions in the brain, fulfilling the second condition for identifying the task-specific interactions of interest.

Subject-level analyses for the generalized nPPI were run using FEAT and implementing FMRIB’s Local Analysis of Mixed Effects (FLAME 1), and examined activation across runs within each participant. Group-level analyses included each subject’s demeaned βs from the prior stimulus-category regressor (see above, Behavioral analysis); this allowed us to examine whether network coupling predicts the characteristics of subsequent RT, and fulfill the third condition for identifying task-specific interactions of interest. The group-level analysis additionally included the main effect of group and three motion-related parameters (SFNR, volume-to-volume head motion, and number of motion spikes within the time series). All resulting z-statistic images were thresholded using a cluster-forming threshold of 2.3 and a corrected cluster-significance threshold of *p* < 0.05. Although this threshold combined with FSL’s FLAME 1 protects against false positives, we note that all of our results also survived permutation-based testing (Eklund et al., 2016). In these supplemental tests, statistical significance was assessed in a nonparametric fashion via FSL’s randomise; this tool uses Monte Carlo permutation-based testing with 10 000 permutations and α = 0.05, corrected for multiple comparisons across the whole brain (Nichols and Holmes, 2002; Winkler et al., 2014).

Brain images and activations are displayed using MRIcroGL (http://www.mccauslandcenter.sc.edu/mricrogl/; Rorden et al., 2007). All coordinates are reported in MNI space.

## Results

### Previous stimulus category influences current behavior

**Figure 2.**
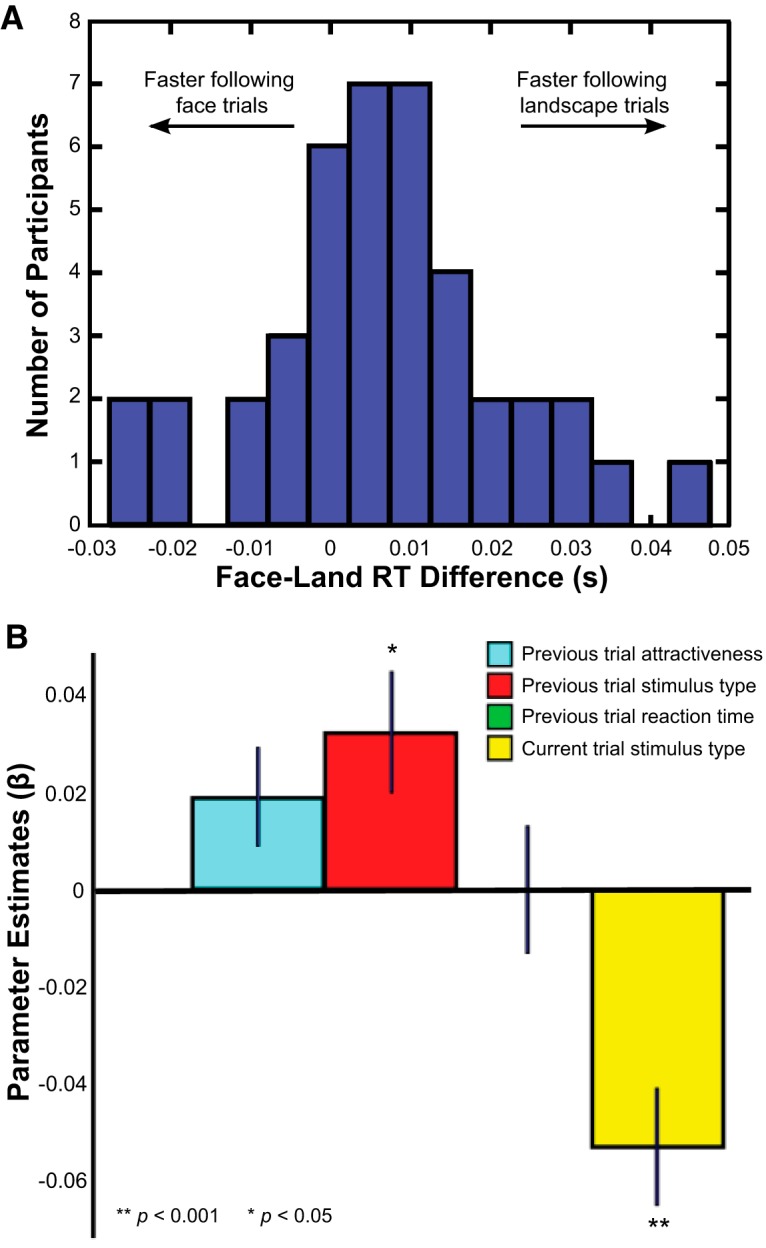
RTs are slower following face trials compared to landscape trials. ***A***, Distribution of RT differences according to previous trial’s stimulus type, calculated by subtracting RTs following nonsocial trials from RTs following social trials. RTs on trials following social rewards were greater than those on trials following nonsocial rewards, indicating an effect of previous reward stimulus type on subsequent behavior. ***B***, Average β weights (with SEM plotted) across subjects from a behavioral regression predicting current RT. We regressed current trial RT on a model including the following regressors: stimulus category (social or nonsocial) on previous trial, RT on previous trial, attractiveness rating of the previous trial’s reward, and stimulus category on current trial. Of these four regressors, current stimulus category most strongly predicted current RT; the negative β weight indicates that participants are faster to respond during a social trial compared to during a nonsocial trial. The next most predictive regressor was previous trial’s stimulus category; the positive β weight indicates that participants were slower to respond following social trials compared to following nonsocial trials. Neither previous trial’s RT nor previous trial’s reward attractiveness significantly predicted the current trial’s RT.

Social rewards, such as images of individuals or interactions with others, provide a useful tool for examining the effects of both social context and nonconsumable rewards on motivated behavior. However, relatively little is known about the effects of social and nonsocial rewards on future motivated behavior, and how the brain orchestrates future motivated action. To examine the effect of social rewards on subsequent motivated actions, we calculated a subsequent-RT effect by subtracting RTs following nonsocial trials from RTs following social trials. When averaging across value and delay trials, we found an overall effect of previous trial stimulus type on current trial RT: participants exhibited increased (slower) RTs subsequent to social trials (M = 0.313 s, SD = 0.008 s) compared to subsequent to nonsocial trials (M = 0.306 s, SD = 0.008 s; *t*_(40)_ = 2.63, *p* = 0.01, *d* = 0.41; Fig. 2*A*). This pattern replicated when examining hit trials only (social: M = 0.317 s, SD = 0.009 s; nonsocial: M = 0.309 s, SD = 0.008 s; *t*_(40)_ = 2.63, *p* = 0.01, *d* = 0.41). Thus, participants were slower after performing a social trial compared to a nonsocial trial.

We next ran a GLM to control for properties of the previous and current trials (see Materials and Methods, Behavioral analysis). Across participants, current stimulus category was the strongest predictor of current RT (*t*_(40)_ = −4.31, *p* < .0001, *d* = 0.67), reflecting that participants were faster to respond during a social trial than during a nonsocial trial. The next strongest predictor was the previous trial’s stimulus category (*t*_(40)_ = 2.46, *p* = 0.01, *d* = 0.38), reflecting that participants were slower to respond following a social trial than following a nonsocial trial. Neither previous trial’s RT nor previous trial’s reward attractiveness rating significantly predicted the current trial’s RT (Fig. 2*B*; Table 1). We note that the effect of previous stimulus type on current RT was still significant even after accounting for the large effect of current stimulus type, reflecting a distinct role of previous stimulus type unaccounted for by the other variables included in the GLM. These results indicate that of the measured properties of the previous trial, prior stimulus type had the strongest effect on current RT.

**Table 1.** Behavioral regression results

Regressor	Parameter estimate (SEM)	*t* stat	*p* value
Current trial (*t*) stimulus type	−0.055 (0.013)	−4.31	<0.0001
Prior trial (*t*-1) stimulus type	0.033 (0.013)	2.46	0.01
Prior trial (*t*-1) attractiveness	0.019 (0.011)	1.74	0.09
Prior trial (*t*-1) RT	<0.0001 (0.013)	0.005	0.99

To examine the effects of other trial characteristics on current trial RT, we regressed current trial RT on a model including: stimulus category (social or nonsocial) on previous trial, RT on previous trial, attractiveness rating of the previous trial’s reward, and stimulus category on current trial. Our analysis indicated that current stimulus type had the strongest effect on current RT; however, of all the characteristics from the prior trial, only prior stimulus type had a significant effect on current RT.

### Network coupling with FG tracks effect of stimulus category on subsequent behavior

Our behavioral results indicated that current-trial stimulus type influences motivated behavior on the subsequent trial (which occurred 7 s or more later), such that participants were slower to respond following social trials compared to following nonsocial trials. However, it remains unclear how interactions in the brain during this previous stimulus outcome affect the subsequent RT. To investigate this, we examined how effective connectivity with the ECN, a network implicated in cognitive control and goal-directed behavior, and the DMN, a network linked with task engagement orchestrate this change in future motivated behavior. We predicted that this subsequent-RT effect would be guided, in part, by changes in the coupling between these large-scale functional networks and domain-specific brain regions when viewing social images compared to when viewing nonsocial images. In particular, we predicted that the subsequent-RT effect would be driven by changes in the relative coupling of the ECN and the DMN during the reward outcome phase of the previous trial. Our analysis pipeline allowed us to meet the criteria to claim that interactions between large-scale networks and specific brain regions are critical for behavior changes (see Introduction).

**Figure 3.**
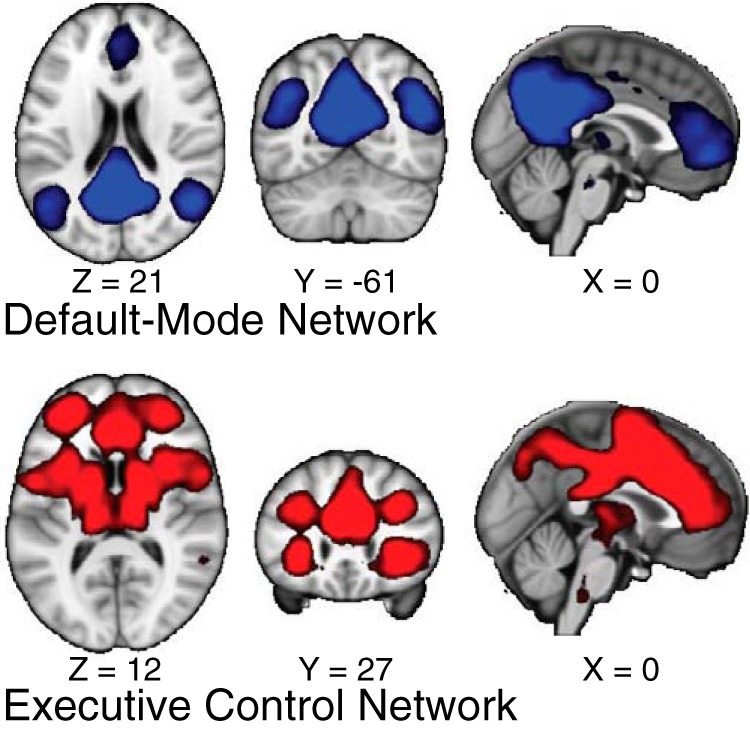
Networks identified from ICA. Using ICA across our task runs, we identified large-scale functional networks. This analysis produced 25 components. Our analyses focused on the components that best matched DMN (highest-correlating component illustrated on top) and the ECN (highest-correlating component illustrated on bottom) from Smith et al. (2009). For visualization purposes, maps are thresholded at Z > 4.

After running the ICA, we identified the DMN and ECN maps by running spatial correlations between the unthresholded maps from our ICA and the DMN and ECN maps from Smith et al. (2009). From our 25 components, we selected the maps that best matched the DMN (*R* = 0.776; other components: R_mean_ = −0.006, R_min_ = −0.179, R_max_ = 0.124) and ECN (*R* = 0.64; other components: R_mean_ = 0.019, R_min_ = −0.092, R_max_ = 0.296) maps from Smith et al. (2009; Table 2). For ease of visualization, thresholded maps (Z > 4) are shown in Figure 3.

**Table 2.** Spatial correspondence with canonical networks

Canonical network	Independent component number	Spatial correlation (*r*)
Visual 1	IC10	0.82
Visual 2	IC01	0.66
Visual 3	IC01	0.45
Default mode	IC06	0.78
Cerebellar	IC18	0.32
Sensorimotor	IC13	0.59
Auditory	IC14	0.66
Executive control	IC04	0.64
R frontoparietal	IC03	0.64
L frontoparietal	IC07	0.77

To identify the DMN and ECN, we ran spatial correlations between canonical neural networks (Smith et al., 2009) and the 25 components from our ICA. The highest-correlating ICA component numbers for each network map and the correlation values are listed.

**Figure 4.**
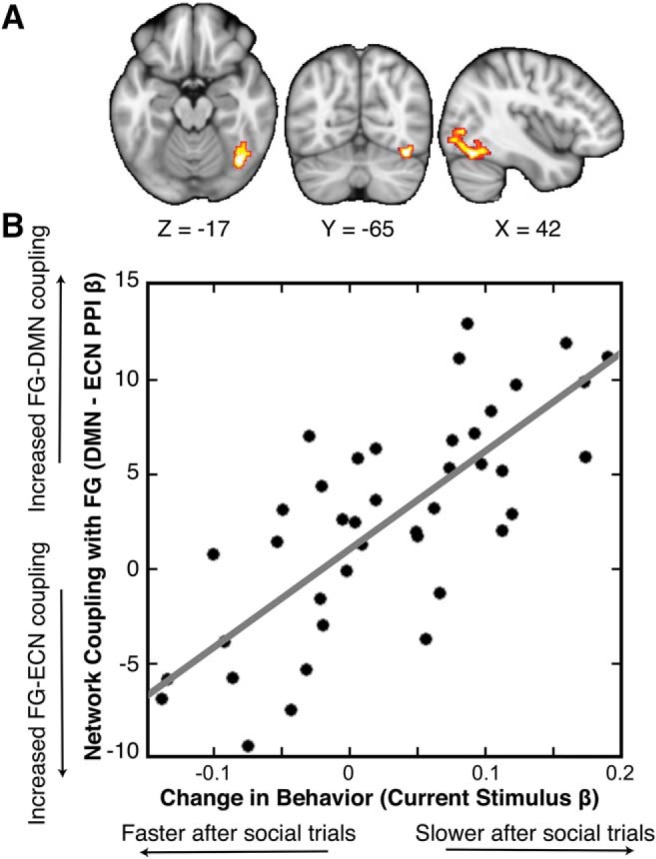
FG connectivity tracks effect of stimulus type on subsequent behavior. ***A***, A nPPI indicated that an area in the FG exhibits heightened effective connectivity with ECN (compared to DMN) during social reward outcomes; thresholded at *p* < 0.05. We note that this result also held with permutation-based testing (Eklund et al., 2016). ***B***, Parameters estimates extracted from the FG connectivity track the effect of stimulus type on subsequent behavior (stimulus-type β weights estimated from our behavioral GLM; see Materials and Methods, Behavioral analysis): as FG-DMN connectivity increases relative to FG-ECN connectivity, RTs are further slowed following social trials compared to following nonsocial trials.

Following network identification, we ran a nPPI analysis that used participant-specific DMN and ECN time courses (estimated by the spatial regression analysis) as the physiologic regressors and presentation of social and nonsocial images as the psychological regressors. This nPPI identified regions that are influenced by the ECN and DMN in a task-dependent manner. We then tested whether these influences on cortex predicted the effect of prior stimulus type on current RT. Our nPPI analysis indicated that effective connectivity between the FG (peak: *x* = 38, *y* = −64, *z* = −20, *p* < 0.0001, voxel extent = 383) and the ECN increased (compared to FG-DMN connectivity) when participants viewed social rewards (Fig. 4*A*), and that the magnitude of this increase tracked the slowing of RT on the subsequent trial (Fig. 4*B*). Strikingly, this peak voxel is consistent with the often functionally-defined FFA (McCarthy et al., 1997; Kanwisher and Yovel, 2006). Identification of the terms associated with this peak voxel using the meta-analytical tool Neurosynth (http://neurosynth.org; Yarkoni et al., 2011) yielded “faces,” “FFA,” and “fusiform face” within its top four associations. These results suggest that functional networks associated with goal-directed and preparatory behavior can interact with focal brain regions to support task-relevant behavior.

To ensure that this connectivity was associated with the previous trial’s outcome rather than the current trial’s cue, we ran an additional nPPI to examine whole-brain connectivity during social cues, nonsocial cues, and nonsocial > social cues. Notably, there were no regions exhibiting changes in connectivity with the ECN relative to the DMN in any of the three contrasts tested. To further investigate this, we used the FG region showing changes in ECN-DMN connectivity during the previous outcome phase as a mask during the current cue phase, and examined whether the ECN-DMN connectivity from this ROI tracked the effect of prior stimulus on current RT. Across the three contrasts tested (social, nonsocial, nonsocial > social), there was no correlation between the ROI connectivity estimates and the effect of previous stimulus type on current RT (social: *r* = −0.12, *p* = 0.45; nonsocial: *r* = 0.01, *p* = 0.94; nonsocial > social: *r* = 0.23, *p* = 0.15). Collectively, these results support the claim that changes in subsequent RT are associated with differential network connectivity with the FG during the previous outcome phase, and cannot be attributed to other aspects of task performance.

No regions showed increased coupling with ECN (relative to DMN) during nonsocial rewards that tracked the effect on subsequent RT, nor did any regions show increased coupling with DMN (relative to ECN) during social or nonsocial rewards that tracked the effect on subsequent RT. Additionally, there were no regions that exhibited changes in coupling with the DMN or ECN when comparing social and nonsocial rewards (e.g., DMN-social > DMN-nonsocial; ECN-social > ECN-nonsocial; and the inverse contrasts) that tracked the effect on subsequent RT. Lastly, we ran a whole-brain GLM using the RT βs as a covariate to examine whether any regions’ activation tracked the effect of stimulus type on subsequent RT; we found that no regions tracked this effect using this traditional GLM. Thus, coupling between large-scale networks and the FG that tracked the effect on subsequent RT was only observed during the viewing of social rewards.

## Discussion

Recent neuroscience research has highlighted the relevance of large-scale functional networks to various aspects of behavior (Eichele et al., 2008; Kelly et al., 2008; Anticevic et al., 2010; Rosen et al., 2016). While many of these studies have linked network activation and connectivity to behavior, the contribution of these networks to motivated behaviors via focal cortical regions has been relatively understudied. For example, although previous work has found correlations between DMN and working memory (Sambataro et al., 2010; Piccoli et al., 2015) or sustained attention (Bonnelle et al., 2011; Gui et al., 2015), understanding how these distributed functional networks influence other cortical regions to shape behavior has remained a significant challenge. Here, we found that participants were slower in a RT task after having performed a trial for a social reward relative to a nonsocial reward (after accounting for the influence of the current trial cue type), reflecting a change in motivated behavior according to previous social stimulus type (Clithero et al., 2011). We then examined the neural mechanisms underlying this effect using nPPI analysis, an adaptation of generalized PPI analysis (i.e., effective connectivity) that examines effective connectivity with large-scale functional networks. This analysis pipeline allowed us to identify changes in coupling between large-scale networks and focal brain regions that predicted subsequent motivated behavior. Our results demonstrate that two goal-relevant networks, the DMN and ECN, interact with FG in a manner that predicts trial-to-trial adjustments in RT.

Our analysis on our modified MID task identified that participants exhibit slower reaction times on trials subsequent to social outcomes, relative to trials subsequent to nonsocial outcomes. These results are consistent with a difference in motivational processes due to the social nature of the previous reward outcome, analogous to effects on motivation observed in similar tasks with nonsocial stimuli (Clithero et al., 2011). There are at least two potential explanations for this subsequent reaction time effect. The first hypothesis is that social images are more distracting than nonsocial images, and participants may still covertly attend to a prior social reward more so than they do a prior nonsocial reward. Previous research indicates that social images interfere with visual attention to a greater degree than nonsocial images (Ebitz et al., 2013); thus, it is possible that this differential interference may affect subsequent trials differently, as well. A second and related potential explanation is that participants’ decreased motivation can be attributed to the satisfaction of receiving a motivating reward on the previous trial, much akin to results seen in “satisfaction of search” (SOS) research. In SOS research, participants performing visual search tasks tend to discontinue their search after finding an initial item, and either miss or display slower reaction times for subsequent items (Berbaum et al., 1990; Fleck et al., 2010). As applied to our MID task, a more motivating reward outcome (social images) may hinder the performance on the subsequent trial. Future research will be needed to explore the cognitive mechanisms driving the observed subsequent-RT effect and to determine if it is specific to social versus nonsocial stimuli, or may generalize to items that are more motivating versus less motivating.

Our findings expand on recent research examining the relevance of DMN and ECN activation to behavior, potentially in task-relevant contexts. Specifically, we found that increased ECN (relative to DMN) coupling with the FG is associated with enhanced subsequent task performance. These results are consistent with previous studies demonstrating different relationships between the DMN and ECN with task behavior: while ECN activation is often associated with heightened task performance and behavior (Dosenbach et al., 2007; Seeley et al., 2007), DMN activation is frequently linked to decrements in behavior and engagement in both humans (Weissman et al., 2006; Eichele et al., 2008) and nonhuman primates (Hayden et al., 2009, 2010; Heilbronner and Platt, 2013). Importantly, however, our findings extend these previous results by demonstrating direct coupling of these networks with a prototypical social-perception processing region in a task-dependent manner. Our results additionally highlight that the opposing effects of DMN and ECN are not limited to concurrent behavior, but also affect subsequent behavior with effects observed seven or more seconds later. These findings support the idea that large-scale networks interact with lower-level perceptual regions to contribute to motivational processes (Clithero et al., 2011) and shape later behavior.

Unlike previous studies examining large-scale networks (Seeley et al., 2007; Brewer et al., 2011; Ossandon et al., 2011; Utevsky et al., 2014), our experiment demonstrates network coupling that directly shapes subsequent RT. While prior studies report associations between functional connectivity and behavior, estimates of functional connectivity solely report on correlations in activation (not coupling) between regions, which can be the result of various phenomena (Friston, 2011). Specifically, reported changes in functional connectivity can arise from numerous causes, including: changes in connectivity with another region, changes in observation noise (or signal-to-noise ratios), and changes in the degree of neuronal fluctuations (for a review, see Friston, 2011). Thus, changes in functional connectivity may not reflect changes in coupling between cortical regions. In contrast to functional connectivity, we implemented an adaptation of a traditional PPI analysis to measure effective connectivity between functional networks and other cortical regions. PPI analyses measure whether a psychological context (e.g., outcome stimulus category) influences how one brain region or network (the “seed”) contributes to another (the “target”) by examining whether an interaction between the psychological context and the seed is identified in the target (Smith et al., 2016). Thus, a PPI analysis can eschew the potential confounds of functional connectivity analyses and reflects a change in neural coupling (e.g., effective connectivity). We note that our novel approach of generalized nPPI, applying generalized PPI analyses to large-scale networks, allows us to examine task-dependent contributions between networks and other cortical regions (Friston et al., 1997; Friston, 2011). In this way, our study extends prior work by demonstrating specific task-dependent coupling of the DMN/ECN.

Although recent meta-analytic work has demonstrated that PPI produces consistent and specific patterns of connectivity (Smith et al., 2016; Smith and Delgado, 2017), it is important to note that PPI results can be interpreted in two ways (Friston et al., 1997). First, our effects could reflect a context-specific modulation of effective connectivity. In this case, face presentations modulate the degree to which the DMN and ECN contribute to FG. Our results focus on the difference between DMN and ECN contributions to FG which seem to facilitate social motivation (Clithero et al., 2011). Alternatively, our effects could reflect a modulation of stimulus-specific responses. In this case, the DMN and ECN influence how FG responds to the presentation of the face; under this interpretation, our results suggest that the degree to which DMN enhances face responses in FG is greater than that of ECN. In either interpretation, the resulting effect on FG connectivity predicts behavior. A better understanding of the mechanisms and causal relationship underlying our results may be facilitated by other analytical approaches, such as dynamic causal modeling (DCM; Friston et al., 2003; Friston, 2011), although relatively less is understood regarding how the biophysical models implemented in DCM apply to distributed functional networks (Buxton et al., 1998; Friston et al., 2003). Additionally, other methodological approaches, such as transcranial magnetic stimulation (Fox et al., 2012; Luber and Lisanby, 2014; Mueller et al., 2014) or transcranial current stimulation (Keeser et al., 2011; Hampstead et al., 2014) may also better inform the specific interactions between FG and the ECN and DMN.

One potential caveat to note is that our task design included a cue to indicate the current trial’s stimulus type in between the previous trial’s outcome phase and the current trial’s response phase. Because the current trial’s stimulus type is the strongest predictor of the current trial’s RT, there may be concerns over whether the neural coupling we observe can be attributed to the cue, rather than the previous trial’s outcome. This concern is ameliorated through both the randomization of trial types (i.e., the identity of the outcome on the previous trial is uncorrelated with the trial type on the subsequent trial), as well as the run-level models implemented in our PPI analysis that included separate regressors for both the outcome phase (social and nonsocial outcomes, separately) and the cue phase (social and nonsocial cues, separately). Including these separate regressors for each phase allowed us to distinguish effects of the cue and outcome phases on connectivity. This concern is additionally mitigated by the analyses examining connectivity during the cue phase. A nPPI showed no changes in ECN-DMN connectivity during social cues, nonsocial cues, or social > nonsocial cues when looking across the whole brain. A supplementary ROI analysis using the FG region indicated that FG connectivity with the ECN (relative to the DMN) during these three contrasts did not track the effect of the previous trial stimulus type on subsequent RT (as illustrated by the lack of significant correlations between the connectivity estimates and the effect of previous stimulus type on subsequent RT). However, future research may benefit from a modified task design in which there are no informative stimuli presented between a reward image and subsequent response.

As an additional caveat, we note that our paradigm and results leave room for interpretive challenges. Because participants exhibited faster RTs to view social images compared to nonsocial images, we cannot discern whether our nPPI results from the social-stimuli condition are due to the stimulus type itself (i.e., face images) or would generalize to other highly motivating stimuli. While prior work linking FG to face processing supports the interpretation that our results are indeed due to that specific stimulus type (Kanwisher et al., 1997; McCarthy et al., 1997; Kanwisher and Yovel, 2006; Engell and McCarthy, 2014), future studies could compare within-domain images of varying attractiveness to other categories of motivating stimuli (e.g., money). Such an analysis would speak to whether these behavioral and neural results are unique to face image rewards, or are due to participant differences in subjective value (Bos et al., 2013; Smith et al., 2014a).

Our results demonstrate task-dependent contributions between the DMN and ECN and the FG that shape subsequent motivated behavior. These large-scale networks are known to be disrupted in a variety of psychopathologies marked by impairments in attention and reward processing, including autism spectrum disorder (Assaf et al., 2010; Young et al., 2015), obsessive compulsive disorder (Stern et al., 2011, 2012), and major depressive disorder (Sheline et al., 2009; Grimm et al., 2011), and so an improved understanding of how they influence moment-to-moment behavior could have clinical relevance and advance models of pathophysiology (Insel et al., 2010; Cuthbert and Insel, 2013). Thus, this study marks a significant step toward better understanding and treatment of disorders characterized by impaired social and reward processing.

## Synthesis

Reviewing Editor: Philippe Tobler, University of Zurich Decisions are customarily a result of the Reviewing Editor and the peer reviewers coming together and discussing their recommendations until a consensus is reached. When revisions are invited, a fact-based synthesis statement explaining their decision and outlining what is needed to prepare a revision will be listed below. The following reviewer(s) agreed to reveal their identity: David Cole.

The authors have addressed the majority of the reviewers' concerns satisfactorily. However, a couple of issues remain. The first one pertains to the most major point raised: The authors highlight in the rebuttal that their analysis has enabled them to “separate the changes in connectivity associated with the cue separately from those associated with the previous outcome”. The logical follow-on question would then ask what (if any) changes in connectivity can be found that are related to the current cue? If, for example, a very similar area of fusiform cortex was identified in an analysis applying the same rigour to investigating current trial cue-related connectivity changes as that which has been reported for previous trial outcome-related changes, this would make the current interpretations positing a selective, time-dependent neurobiological mechanism rather problematic (despite the lack of collinearity between the task measures). Results of this additional analysis would help to resolve this question (a) if there is/is not a spatially similar or dissociable relationship for cue-related region-network connectivity, (b) if any such finding is stronger/weaker than the outcome-related connectivity and (c) if the association of this connectivity with RT behaviour is greater/lesser than the reported association for outcome.

Secondly, the proposed mechanisms, buried in the Discussion, should at the very least be introduced early in the manuscript. Readers will likely be mystified that the influence of the trial cue is not fully considered (it isn't included in the PPI statistical model). Explaining that the authors are not looking for indicators of social motivation (which would be strongly linked to the cue), but to potential sources of social interference (see Discussion), will go a long way to setting the stage for the proper context of these findings.

Thirdly, some areas of text still need to have language cleaned up regarding the nature of the RTs. For example, in the discussion: “Here, we found that participants were slower in a response time task after having performed a trial for a social reward relative to a non-social reward, reflecting a change in motivated behavior according to previous social stimulus type (Clithero et al., 2011).” Subjects had faster RTs for social cue trials, and once that was accounted for, there was a residual influence of previous trial outcome. Statements like this are somewhat misleading, and the findings is actually quite different than the cited Clithero paper.

## Author Response

Response to Reviewers

Significance Statement Comments for Authors:

The reviewers were happy with the methodological approach and the proposition of a new mechanism of attentional interference to be considered in addition to social motivation. However, they criticized that the reader won't know that until the discussion.

We thank the Reviewers for their constructive feedback. We have made the changes recommended by the Reviewers and believe our manuscript has been strengthened in doing so. We have addressed the Reviewers' concerns below.

Synthesis Statement for Authors:

The authors have addressed the majority of the reviewers' concerns satisfactorily. However, a couple of issues remain. The first one pertains to the most major point raised: The authors highlight in the rebuttal that their analysis has enabled them to “separate the changes in connectivity associated with the cue separately from those associated with the previous outcome”. The logical follow-on question would then ask what (if any) changes in connectivity can be found that are related to the current cue? If, for example, a very similar area of fusiform cortex was identified in an analysis applying the same rigour to investigating current trial cue-related connectivity changes as that which has been reported for previous trial outcome-related changes, this would make the current interpretations positing a selective, time-dependent neurobiological mechanism rather problematic (despite the lack of collinearity between the task measures). Results of this additional analysis would help to resolve this question (a) if there is/is not a spatially similar or dissociable relationship for cue-related region-network connectivity, (b) if any such finding is stronger/weaker than the outcome-related connectivity and (c) if the association of this connectivity with RT behaviour is greater/lesser than the reported association for outcome.

We appreciate this concern and thank the reviewers for the suggestions. To address the question of changes in connectivity related to the current cue, we ran two new analyses:

* First, we ran a whole-brain nPPI analysis looking at the effects of social cues, nonsocial cues, and nonsocial < social cues (as well as the inverse contrast). A whole-brain analysis with permutation testing (implementing threshold-free cluster enhancement with 10,000 permutations) showed no significant change in connectivity associated with the cue epoch for social cues, nonsocial cues, or the difference between social cues and nonsocial cues (all ps < 0.05).

* Second, we ran an ROI analysis using the FG mask extracted from the original PPI analysis. We used this ROI as a mask to extract betas from the social cue, nonsocial cue, and nonsocial cue < social cue contrasts, and examined whether these betas showed a relationship with the reaction time betas used in the original nPPI analysis. None of the nPPI contrast betas exhibited a significant correlation with reaction time betas (social cues: r = -0.12, p = 0.45; nonsocial cues: r = 0.01, p = 0.94; nonsocial < social: r = 0.23, p = 0.15). To illustrate this, we have attached the original scatter plot from the manuscript below, along with the new scatter plots below illustrating the relationships between the connectivity with FG during the various cue epochs. While we have not included these additional figures in the manuscript, we have added the results from the correlation analyses to the Results section.

To illustrate the results of those analyses graphically, we include below the scatterplots for the difference between non-social and social cues (left panel), for social cues (middle panel), and for nonsocial cues (right panel) - each plotted as function of the behavior change on the current trial. As can be seen in these plots, there are no relationships between that cue-related activity and behavior; these can be compared to Figure 4B in the manuscript, which shows a strong relationship between reward-related changes in connectivity and behavior change.

[FIGURE HERE]

We have added the following sections to the manuscript. We believe that these results address the reviewers' concerns.

Results section: “To ensure that this connectivity was associated with the previous trial's outcome rather than the current trial's cue, we ran an additional nPPI to examine whole-brain connectivity during social cues, nonsocial cues, and nonsocial < social cues. Notably, there were no regions exhibiting changes in connectivity with the ECN relative to the DMN in any of the three contrasts tested. To further investigate this issue, we used the FG region showing changes in ECN-DMN connectivity during the previous outcome phase as a mask during the current cue phase, and examined whether the ECN-DMN connectivity from this region of interest (ROI) tracked the effect of prior stimulus on current RT. Across the three contrasts tested (social, nonsocial, nonsocial < social), there was no correlation between the ROI connectivity estimates and the effect of previous stimulus type on current RT (social: r = -0.12, p = 0.45; nonsocial: r = 0.01, p = 0.94; nonsocial < social: r = 0.23, p = 0.15). Collectively, these results support the claim that changes in subsequent RT are associated with differential network connectivity with the FG during the previous outcome phase, and cannot be attributed to other aspects of task performance.”

Discussion: “This concern is additionally mitigated by the analyses examining connectivity during the cue phase. A nPPI showed no changes in ECN-DMN connectivity during social cues, nonsocial cues, or social < nonsocial cues when looking across the whole brain. A supplementary ROI analysis using the FG region indicated that FG connectivity with the ECN (relative to the DMN) during these three contrasts did not track the effect of the previous trial stimulus type on subsequent RT (as illustrated by the lack of significant correlations between the connectivity estimates and the effect of previous stimulus type on subsequent RT).”

Secondly, the proposed mechanisms, buried in the Discussion, should at the very least be introduced early in the manuscript. Readers will likely be mystified that the influence of the trial cue is not fully considered (it isn't included in the PPI statistical model). Explaining that the authors are not looking for indicators of social motivation (which would be strongly linked to the cue), but to potential sources of social interference (see Discussion), will go a long way to setting the stage for the proper context of these findings.

We agree with this suggestion. Our nPPI model does include regressors for the cue phase, which is noted in the Methods section (Lines 262 - 264: “The run-level model included six task regressors: social cue (duration = 1.5 - 4.5 s), nonsocial cue (duration = 1.5 - 4.5 s), hits (duration = 0.75 s), misses (duration = 0.75 s), social reward outcome (duration = 2 s), and nonsocial reward outcome (duration = 2 s).”).

However, we agree that it would be helpful to introduce potential mechanisms underlying this effect in the Introduction. To address this concern, we have added the following text to the Introduction discussing the proposed mechanisms underlying this change in connectivity and its relationship to behavior:

“These findings highlight functional network interactions that guide subsequent changes in reaction time behavior. While the cognitive mechanisms underlying the behavioral effect of previous stimulus type on subsequent reaction time require future research, we propose that this effect may be driven by increased attentional interference of social rewards relative to nonsocial rewards or a subsequent change in task motivation.”

Thirdly, some areas of text still need to have language cleaned up regarding the nature of the RTs. For example, in the discussion: “Here, we found that participants were slower in a response time task after having performed a trial for a social reward relative to a non-social reward, reflecting a change in motivated behavior according to previous social stimulus type (Clithero et al., 2011).” Subjects had faster RTs for social cue trials, and once that was accounted for, there was a residual influence of previous trial outcome. Statements like this are somewhat misleading, and the findings is actually quite different than the cited Clithero paper.

We have added language to our Discussion to qualify this claim, and hope that this addition helps to clarify our interpretations of the results. Additionally, while our findings are different from the Clithero (2011) paper, we cite that paper to support the claim that the MID task - of which our paradigm is a social variant - probes motivation for the trial outcome.
